# The application of tetracyclineregulated gene expression systems in the validation of novel drug targets in *Mycobacterium tuberculosis*

**DOI:** 10.3389/fmicb.2015.00812

**Published:** 2015-08-04

**Authors:** Joanna C. Evans, Valerie Mizrahi

**Affiliations:** ^1^South African Medical Research Council/National Health Laboratory Service/University of Cape Town Molecular Mycobacteriology Research UnitCape Town, South Africa; ^2^DST/NRF Centre of Excellence for Biomedical TB Research, Institute of Infectious Disease and Molecular Medicine and Division of Medical Microbiology, Faculty of Health Sciences, University of Cape TownCape Town, South Africa

**Keywords:** *Mycobacterium tuberculosis*, drug discovery, regulated gene expression, hypomorphs, target validation, target-based whole cell screening

## Abstract

Although efforts to identify novel therapies for the treatment of tuberculosis have led to the identification of several promising drug candidates, the identification of high-quality hits from conventional whole-cell screens remains disappointingly low. The elucidation of the genome sequence of *Mycobacterium tuberculosis* (*Mtb*) facilitated a shift to target-based approaches to drug design but these efforts have proven largely unsuccessful. More recently, regulated gene expression systems that enable dose-dependent modulation of gene expression have been applied in target validation to evaluate the requirement of individual genes for the growth of *Mtb* both *in vitro* and *in vivo*. Notably, these systems can also provide a measure of the extent to which putative targets must be depleted in order to manifest a growth inhibitory phenotype. Additionally, the successful implementation of *Mtb* strains engineered to under-express specific molecular targets in whole-cell screens has enabled the simultaneous identification of cell-permeant inhibitors with defined mechanisms of action. Here, we review the application of tetracycline-regulated gene expression systems in the validation of novel drug targets in *Mtb*, highlighting both the strengths and limitations associated with this approach to target validation.

Tuberculosis (TB) remains a major global health crisis, with 9 million new cases and 1.5 million deaths attributable to this disease in 2013 (WHO, 2014). The treatment of TB is challenging, requiring combination therapy with rifampicin, isoniazid, pyrazinamide, and ethambutol for 2 months, followed by an additional 4 months of treatment with rifampicin and isoniazid. Despite this extended period of treatment, however, strains of *Mycobacterium tuberculosis* (*Mtb*) resistant to rifampicin and isoniazid, defined as multidrug-resistant (MDR), continue to emerge, with an estimated 480,000 cases of MDR-TB identified in 2013 (WHO, 2014). The more recent emergence of MDR strains with additional resistance to fluoroquinolones and at least one of the injectable aminoglycosides (amikacin, kanamycin, or capreomycin) – termed extensively drug-resistant (XDR) – has resulted in the need to implement therapies that must be continued for up to 24 months, and involve the use of more costly, less effective, and more toxic second-line drugs. Thus, the emergence and spread of drug-resistant strains of *Mtb* has complicated the already daunting challenge of global TB control, and has underscored the urgent need to identify new anti-tubercular agents with novel mechanisms of action (Zumla et al., 2013). This need has driven the establishment of a TB drug pipeline, which is populated with a number of promising candidates and novel drug combinations at various stages of clinical development. However, the typically high attrition rate for drugs in clinical development, coupled with specific weaknesses in the TB drug pipeline – in particular, the paucity of candidates in the early stages of clinical validation – makes the development of new drug regimens for the treatment of drug-resistant as well as drug-susceptible TB critically reliant upon replenishment of the pipeline through the identification of high-quality “hit” compounds and novel targets by innovative TB drug discovery programs.

In the target-based approach to TB drug discovery, compounds that inhibit the biochemical function of the target of interest are identified by high-throughput screening (HTS) of compound libraries or by structure-guided drug design (Hung et al., 2009; Willand et al., 2009; Krieger et al., 2012). While this approach has yielded compounds with potent inhibitory activity against validated targets, the compounds frequently fail to display whole-cell activity against live bacilli (Payne et al., 2007; Cole and Riccardi, 2011; Xu et al., 2014). This problem is commonly attributed to issues of permeation, metabolism, and eﬄux, all of which are likely exacerbated by the comparatively complex intracellular environment in which the drug-target interaction must occur in a cell-based assay. In the alternative, phenotypic approach, cell-based HTS of compound libraries has been used to identify inhibitors with whole-cell activity against *Mtb in vitro*. This approach has been considerably more successful (Stover et al., 2000; Matsumoto et al., 2006; Makarov et al., 2009; Pethe et al., 2013; Manjunatha et al., 2015), and led to the recent development of bedaquiline (Sirturo; Andries et al., 2005), the first FDA-approved drug for the treatment of TB in over 40 years (Cohen, 2013); delamanid (Deltyba), which received conditional approval by the European Medicines Agency in 2013 and is being evaluated in a phase 3 trial for the treatment of MDR-TB (Gler et al., 2012); and PA-824 (pretomanid), which has shown promise as part of a new three-drug regimen (Dawson et al., 2015). Importantly, hit compounds discovered through phenotypic screens have been used to identify novel targets in previously unexploited pathways, such as AtpE (target of bedaquiline) and QcrB (targeted by imidazopyridines other chemotypes) in energy metabolism, and DprE1 (benzothiazinones and other chemotypes) in arabinan biosynthesis (Andries et al., 2005; Petrella et al., 2006; Makarov et al., 2009; Magnet et al., 2010; Abrahams et al., 2012b; Stanley et al., 2012; Pethe et al., 2013; Preiss et al., 2015). However, deducing mechanisms of action of inhibitors with whole-cell activity can often be challenging, particularly if the inhibitory effects are pleiotropic (Kohanski et al., 2008) or if the target is not a protein (Ling et al., 2015).

A fundamental requirement of a desirable antimicrobial drug target is that it should have an essential, and preferably non-redundant, function in growth and pathogenesis of the organism. The study of (conditionally) essential gene function in bacteria has been revolutionized by the development of regulated gene expression systems, which enable the expression of a target gene to be controlled in a manner that depends upon the level of an inducer. A number of such systems have been developed for use in mycobacteria, and have been employed to investigate the roles of a wide variety of genes essential and conditionally essential for bacillary growth, physiology, and metabolism (Gomez and Bishai, 2000; Greendyke et al., 2002; Dziadek, 2003; Trauner et al., 2012; Ventura et al., 2013; Chuang et al., 2015). In this review, we focus on the use of tetracycline (Tet)-regulated gene expression systems in *Mtb*, specifically in the context of target validation. We highlight the technical advancements that have been made, and describe the application of conditional mutants of *Mtb* in the validation of novel drug targets, and in target-based whole-cell screening (TB-WCS), in which phenotypic and target-based approaches are combined to identify target- and pathway-selective compounds with whole-cell activity.

## Tet-Regulated Genetic Switches for Controlling Gene Expression in *Mtb*

Regulated gene expression systems have been extensively characterized and utilized in both Gram-positive and Gram-negative bacteria (Bertram and Hillen, 2008), yet limited knowledge of the transcriptional regulatory mechanisms and machinery complicated the development of such systems for use in mycobacteria until relatively recently. The first successful demonstration of dose-responsive gene expression in mycobacteria was achieved by expressing the 35 kDa antigen of *M. leprae* from the acetamide-inducible promoter of *Mycobacterium smegmatis* (*Msm*; Parish et al., 1997; Triccas et al., 1998). This system has since been used successfully to express several mycobacterial proteins in *Msm* (Gomez and Bishai, 2000; Ojha et al., 2000; Parish et al., 2001; Daugelat et al., 2003; Dziadek, 2003; Ahidjo et al., 2011; Lee et al., 2014); however, instability in *Mtb* has limited its utility in this species (Brown and Parish, 2006). Several other regulated gene expression systems, including those mediated by Tet, nitrile, and pristinamycin, have subsequently been developed for use in mycobacteria (**Table 1A**); these have been reviewed elsewhere (Schnappinger and Ehrt, 2014) so will not be described here. In this review, we focus specifically on the Tet-regulated gene expression systems (**Table 1B**; Blokpoel et al., 2005; Carroll et al., 2005; Ehrt et al., 2005), which have been widely applied, and whose utility in TB drug discovery is enhanced by the fact that they can be used to regulate gene expression in animal models of infection.

**Table 1:** Regulated gene expression systems developed for use in *Mycobacterium tuberculosis* (*Mtb*).

Expression system	Regulatory components		Reference
	Promoter	Regulator	
**(A) Gene regulation systems utilized in mycobacteria**	
Tet-ON	Tet-inducible promoter of *tetA from Corynebacterium glutamicum*	TetR repressor from *C. glutamicum*	Blokpoel et al. (2005)
Tet-ON	Tet-inducible promoter P_xyl_ from *Bacillus subtilis*	Tn*10*-derived TetR repressor from *Escherichia coli*	Carroll et al. (2005)
NitR	Nitrile-inducible promoter of *nitA* from *Rhodococcus rhodochrous*	NitR regulator, both from *R. rhodochrous*	Pandey et al. (2009)
Pip-ON	Pristinamycin-inducible promoter, P*_ptr_*, from *Streptomyces pristinaespiralis*	Pristinamycin-responsive repressor, Pip, from *S. coelicolor*	Forti et al. (2009)
Tet/Pip-OFF	Pristinamycin-inducible promoter, P*_ptr_*, from *S. pristinaespiralis*	Pristinamycin-responsive repressor, Pip, from *S. coelicolor* under control of Tet-inducible promoter, P_furA102_*tetO*	Boldrin et al. (2010)
**(B) Tetracycline (Tet)-regulated gene expression systems optimized for use in mycobacterial gene regulation**			
Tet-ON	Tet-inducible mycobacterial promoter, P_myc1_*tetO*	Tn*10*-derived TetR repressor from *E. coli*	Ehrt et al. (2005)
Tet-OFF	Tet-inducible mycobacterial promoter, P_myc1_*tetO*	Tn*10*-derived reverse TetR inducer from *E. coli*	Guo et al. (2007)

**FIGURE 1:**
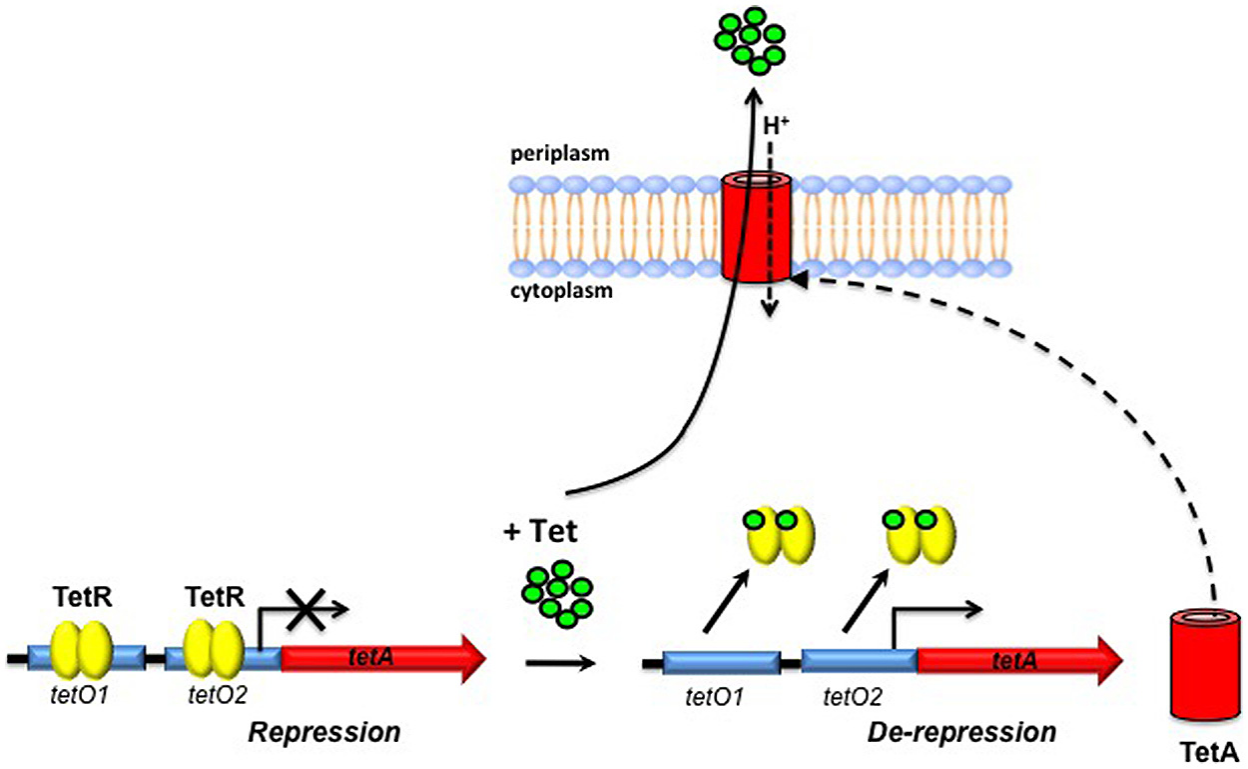
**Schematic representation of tetracycline (Tet) export in Gram-negative bacteria**. In the absence of Tet, Tet repressor (TetR) dimers bind to Tet operators (*tetO*) located upstream of *tetA*, encoding the Tet-exporting protein, repressing its transcription. Upon introduction of anhydrotetracycline (ATc) and its subsequent binding to TetR, a conformational change occurs that results in dissociation of TetR from *tetO*, enabling Tet-mediated transcription of *tetA* to occur. Tet is then transported across the cytoplasmic membrane by TetA.

The mechanism of transcriptional regulation using Tet-controlled genetic switches is based on that of a family of Tet-exporting proteins that mediate resistance to Tet in Gram-negative organisms (Berens and Hillen, 2003). In the absence of Tet, Tet repressor (TetR) proteins bind tightly to the Tet operators (*tetO*) located within the promoter of *tetA*, encoding the Tet-exporting protein, thereby suppressing *tetA* expression (**Figure 1**; Berens and Hillen, 2003). Owing to the substantially higher affinity of TetR for Tet than for the ribosome (Lederer et al., 1996), the introduction of Tet causes a conformational change that results in dissociation of TetR from *tetO*, leading to de-repression of *tetA* and ultimately to the eﬄux of Tet (Berens and Hillen, 2003). By replacement of the native *Mtb* promoter of a gene of interest with a Tet-regulatable promoter, and introduction of TetR on a replicating or integrative plasmid, dose-dependent induction of gene expression can be mediated through the addition of varying concentrations of the less toxic derivative, anhydrotetracycline (ATc; Blokpoel et al., 2005; Carroll et al., 2005; Ehrt et al., 2005). Since it is important that introduction of the inducer has a minimal effect on the expression of other genes, an additional advantage of the Tet-regulated system is that gene expression can be induced with ATc concentrations at least 10-fold below the concentration required to inhibit the growth of *Msm* or *Mtb* (Ehrt and Schnappinger, 2006).

**FIGURE 2:**
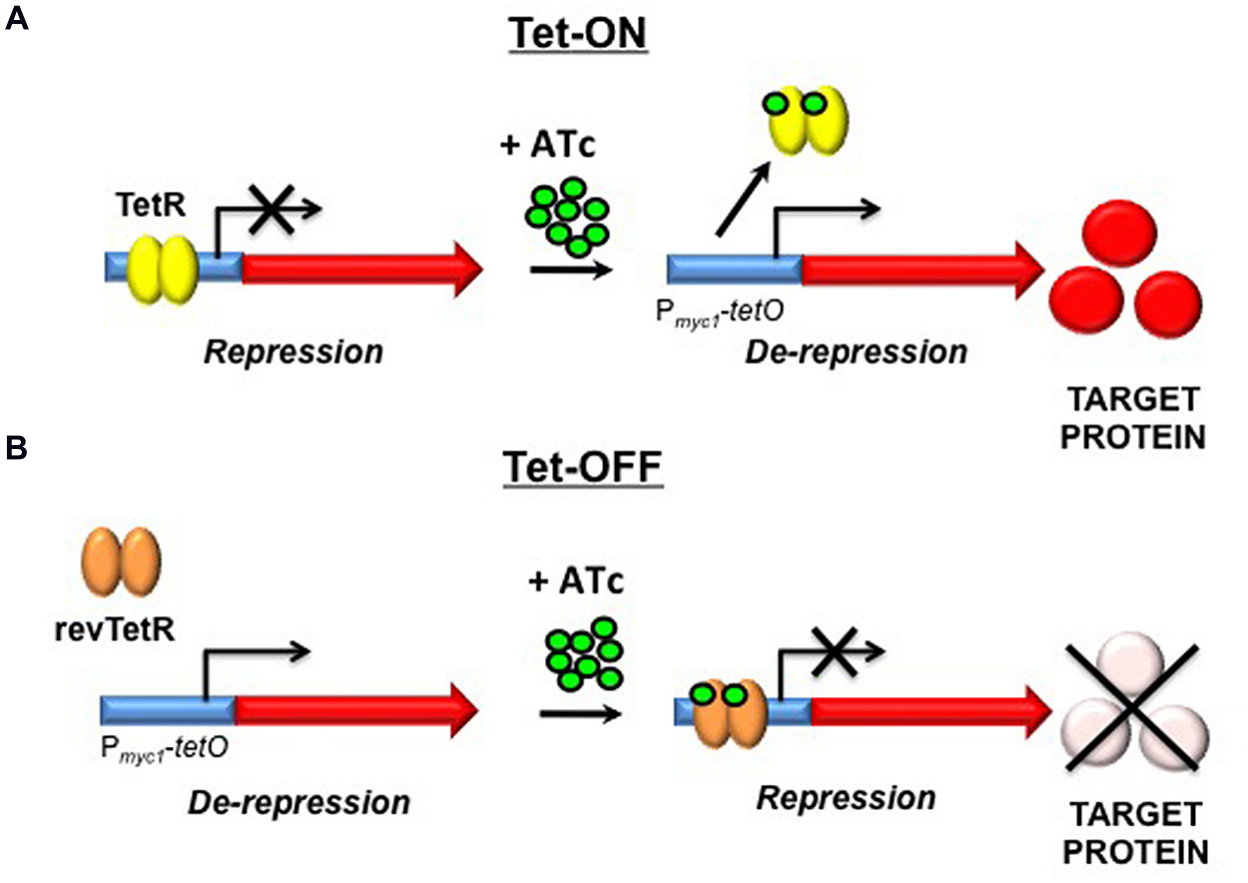
**Tetracycline-mediated gene regulation in *Mycobacterium tuberculosis* (*Mtb*). (A)** In the Tet-ON configuration, TetR dimers bind to Tet operators (*tetO*) in the absence of ATc, repressing transcription. Upon introduction of ATc and its subsequent binding to TetR, a conformational change occurs that results in dissociation of TetR from *tetO*, enabling Tet-mediated transcription from P_myc1_*tetO* to occur. **(B)** In the Tet-OFF configuration, introduction of ATc results in binding of revTetR to P_myc1_*tetO*, causing repression of transcription. TetR, Tet repressor; ATc, anhydrotetracycline; *tetO*, Tet operator; P_myc1_*tetO*, mycobacterial promoter with *tetO*s inserted; revTetR, reverse Tet repressor (Adapted from Guo et al., 2007).

Conditional knockdown mutants – or “hypomorphs” – in which expression of the target gene is switched on upon addition of ATc are commonly referred to as “Tet-ON” mutants; in this configuration, removal of the inducer is required in order to silence target gene expression (**Figure 2A**). The dynamic range of the Tet-ON system can be expanded further by expression of TetR from either a strong promoter or an intermediate-strength promoter to generate hypomorphs in the Tet-ON_S_ and Tet-ON_M_ configurations, respectively. This increases the flexibility of the system, enabling dose-dependent regulation of genes with widely differing levels of expression. Since the removal of ATc can be difficult to achieve in some experimental settings, the manipulation of Tet-dependent hypomorphs was greatly simplified by the development of a modified “Tet-OFF” system, which utilizes a mutated, “reverse” TetR (revTetR) that binds to *tetO* only in the presence of ATc (Guo et al., 2007). This system enables the generation of mutants in which target gene expression is repressed upon addition of the ATc inducer (**Figure 2B**). The regulatory capacity of both Tet-ON and Tet-OFF systems in mycobacteria has been enhanced by codon optimization of the genes encoding TetR and revTetR to allow increased expression in *Mtb* (Klotzsche et al., 2009). Together, these modifications have provided a means of evaluating the effects of transcriptional silencing of target genes on growth and viability of the organism.

## Application of Tet-Regulated Systems in Target Validation

The utility of genetic switches for regulating gene expression in *Mtb* was first demonstrated by the generation of a tryptophan auxotroph following Tet-mediated transcriptional silencing of *trpD* (Carroll et al., 2005). Transcriptional silencing has subsequently been used to confirm the *in vitro* essentiality of a variety of genes in *Mtb*, thus providing preliminary genetic validation of the encoded proteins as potential drug targets. These include, among others, fatty-acid-coenzyme A (CoA) ligase (*fadD32*; Carroll et al., 2011), diaminopimelate decarboxylase (*lysA*; Abrahams et al., 2012a), Clp protease (*clpP1*; Ollinger et al., 2012b), pantothenate synthetase (*panC*; Abrahams et al., 2012a), thymidylate synthase (*thyX*; Singh et al., 2015), ribonucleotide reductase (*nrdF2*; Singh et al., 2015), topoisomerase I (*Rv3646c*; Ahmed et al., 2014) and seven genes (*dprE1*, *dprE2*, *ubiA*, *prsA*, *Rv2361c*, *tkt*, and *rpiB*) involved in decaprenyl-phospho-D-arabinofuranose (DPA) biosynthesis in *Mtb* (Kolly et al., 2014a).

A major advantage of Tet-regulated systems is that the ability of ATc to diffuse across biological membranes also allows these systems to be used to regulate expression of *Mtb* genes in infected macrophages (Blokpoel et al., 2005; Ehrt et al., 2005). Furthermore, the pharmacokinetic properties of ATc and its analog, doxycycline (doxy), permit administration of this inducer to animals at doses required to successfully regulate gene expression *in vivo* (Ehrt and Schnappinger, 2006) without compromising the growth of wildtype bacilli (Gandotra et al., 2007; Blumenthal et al., 2010). Since the conditions under which *Mtb* can be cultured *in vitro* are unlikely to fully recapitulate the environment encountered in the host during infection, this can have significant effects on the physiology, metabolism and global transcriptional response of the bacilli and, hence, on the relevance of the conclusions drawn from such analyses (Bloch and Segal, 1956; Schnappinger et al., 2003; Pethe et al., 2010; Griffin et al., 2012). The development of the Tet-system therefore represented a major breakthrough in mycobacterial genetics by providing a means to identify genes that are (conditionally) essential for growth of *Mtb in vivo* (Gandotra et al., 2007). Silencing of *prcBA*, encoding the α and β subunits of the *Mtb* 20S proteasome, either immediately post-infection, or during the chronic stage of infection (i.e., following onset of the acquired immune response), resulted in a decline in bacillary load in the lungs and spleens of mice, indicating a role for the proteasome in both growth and persistence of *Mtb in vivo* (Gandotra et al., 2007). The successful demonstration of the utility of this approach paved the way for a slew of subsequent studies aimed at investigating the requirement of several *Mtb* genes for growth during different phases of infection (**Table 2**), simultaneously providing genetic validation of many of these as potential drug targets. Although perhaps not of direct relevance to the physiology of *Mtb* itself, it is worth noting that the selective inhibition of mitochondrial protein translation by Tets can lead to altered transcriptional profiles in eukaryotic cell lines and has minor effects on physiological fitness in mice (Moullan et al., 2015). This may, in turn, affect the contribution of the murine immune response to *Mtb* clearance.

**Table 2:** Targets validated by Tet-mediated control of *Mtb* gene expression *in vivo.*

Gene	Protein	Function	Phase of infection	Reference
*prcBA*	Proteasome α and β subunits	ATP-dependent degradation of damaged proteins	Chronic	Gandotra et al. (2007), Blumenthal et al. (2010)
*pckA*	Phosphoenolpyruvate carboxykinase	Catalyzes the first committed step in gluconeogenesis	Acute and chronic	Marrero et al. (2010)
*icl1*	Isocitrate lyase	Catalyzes the conversion of isocitrate to glyoxylate and succinate in the first step of the glyoxylate shunt	Acute and chronic	Blumenthal et al. (2010)
*Rv3671c*	Serine protease	Role in protection from oxidative stress and acidification	Chronic	Blumenthal et al. (2010)
*pptT*	4′-phosphopantetheinyl transferase	Transfer of 4′-phosphopantetheine group from Coenzyme A (CoA) to acyl carrier proteins	Acute and chronic	Leblanc et al. (2012)
*fba*	Fructose-1,6-bisphosphate aldolase (FBA)	Reversible cleavage of fructose-1,6-bisphosphate to yield dihydroxyacetone phosphate and glyceraldehyde 3-phosphate in glycolysis and gluconeogenesis	Acute and chronic	Puckett et al. (2014)
*pimA*	Phosphatidyl-*myo*-inositol mannosyltransferase	Transfer of mannosyl residue from GDP-Man to phosphatidyl-*myo*-innositol	Acute and chronic	Boldrin et al. (2014)
*relA*	GTP-pyrophosphokinase	Synthesis of (p)ppGpp	Acute and chronic	Weiss and Stallings (2013)
*carD*	Transcriptional regulator	Regulates transcription initiation Srivastava et al. (2013)	Acute and chronic	Stallings et al. (2009)
*bioA*	7,8-diaminopelargonic acid synthase	Catalyzes the antepenultimate step in biotin biosynthesis	Acute and chronic	Woong Park et al. (2011)

An important caveat to the use of hypomorphs for confirming target essentiality both *in vitro* and *in vivo* is the potential for suppressors to emerge that are no longer responsive to the inducer (Stallings et al., 2009; Boldrin et al., 2010; Kolly et al., 2014a). The loss of inducer dependence of gene expression can arise through the acquisition of mutations in the regulatory network and/or loss of the transcriptional repressor as a consequence of the strong selective pressure conferred on *Mtb* by depletion of an essential cellular function. Since the emergence of suppressor mutants masks the effects of inducer-dependent transcriptional silencing, it is essential to include appropriate controls for detection of populations of bacilli that are no longer inducer-responsive when phenotypically characterizing hypomorphs produced using these systems.

## Assessing Target Vulnerability by Tet-Mediated Transcriptional Silencing

**FIGURE 3:**
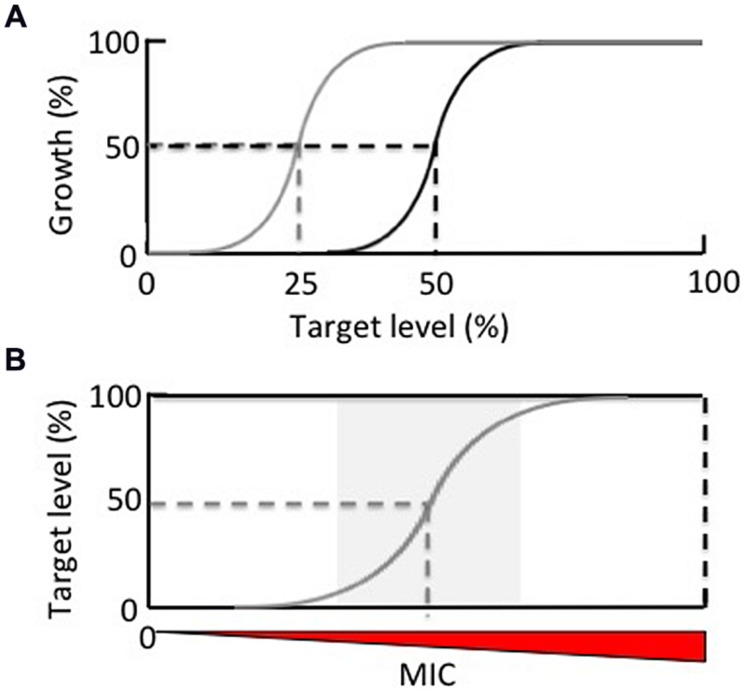
**The application of hypomorphs in **(A)** assessing target vulnerability and **(B)** expanding antitubercular chemical space. (A)** Dose-dependent silencing of a target gene enables determination of the extent to which that target must be depleted in order to result in growth inhibition. A target that requires only 50% depletion in order to reduce 50% of bacterial growth (black line) is considered more vulnerable that one that requires 75% depletion to achieve the same effect (gray line). **(B)** Reduction of the absolute concentration of a target by conditional knockdown (gray line) relative to wildtype levels (black line) facilitates the identification of on-target inhibitors by lowering the minimum inhibitory concentration (MIC).

Genome-wide transposon mutagenesis has played a critical role in defining the genetic requirements for growth and viability of *Mtb* under various conditions (Sassetti and Rubin, 2003; Sassetti et al., 2003; Rengarajan et al., 2005; Beste et al., 2009; Griffin et al., 2011). However, the complete elimination of biochemical function resulting from insertional inactivation of a gene encoding an essential cellular function is unlikely to be achieved by chemical inhibition of that function, particularly in the validation phase of drug discovery when inhibitory activity has not yet been optimized (Barry et al., 2009). Recent target validation efforts have thus focused on determining target “vulnerability,” which can be operationally defined as the extent to which the activity/function of a target must be reduced in order to manifest in a growth phenotype (**Figure 3A**). Given increasing evidence suggesting that targets requiring incomplete inhibition in order to confer a lethal phenotype may be more valuable (Barry et al., 2009; Kim et al., 2011; Wei et al., 2011), the ability to silence gene expression in a dose-dependent manner provides a potentially powerful means to infer the vulnerability of a given target.

The construction of hypomorphs by transcriptional silencing has allowed target vulnerability to be analyzed by quantifying the relative level of transcript depletion associated with bacterial growth inhibition and/or loss of viability (Gandotra et al., 2007; Forti et al., 2009; Boldrin et al., 2010; Carroll et al., 2011; Kolly et al., 2014a). However, the kinetics of depletion of a protein by transcriptional silencing depend upon the specific protein half-life. Therefore, it is important to monitor the extent of protein depletion and its association with bacterial growth and/or viability during the course of a silencing experiment as silencing of genes encoding long-lived proteins may result in an underestimation of the vulnerability of those targets. It is also important to consider the relative strengths of the inducible promoter vs. the native promoter of the gene of interest when constructing hypomorphs. In cases where the target protein is in low abundance in wildtype bacilli, promoter replacement may result in the target being expressed at a higher level from the regulated promoter than from its native promoter under conditions of maximal de-repression. In such cases, efficient silencing is unlikely to be achieved (Ehrt and Schnappinger, 2006; Carroll et al., 2011). The converse may also occur, as observed in the case of *icl1*, which encodes isocitrate lyase, a key enzyme in the glyoxylate shunt. Icl1 is conditionally essential for growth of *Mtb* on fatty acids and is transcriptionally upregulated when the organism is cultured on acetate (Munoz-Elias and McKinney, 2005; Blumenthal et al., 2010). Replacement of the native *icl1* promoter with the Tet-regulated promoter, P_myc1_*tetO*, resulted in a 16-fold increase in sensitivity of *Mtb* to the Icl inhibitor, 3-nitropropionate (3-NP), when grown on acetate, most likely reflecting a significantly lower level of *icl1* expression by the Tet-regulated promoter than the native (fatty-acid-inducible) promoter (Abrahams et al., 2012a).

Transcriptional silencing has been used to infer relative invulnerability for a number of potential drug targets for which small molecule inhibitors have been developed. For example, pantothenate kinase (PanK), the enzyme that catalyzes the first, and rate-limiting, step in the conversion of pantothenate (vitamin B5) into CoA (Das et al., 2006), has attracted considerable attention as a TB drug target (Begley et al., 2001; Spry et al., 2008; Moolman et al., 2014). Although essential for growth of *Mtb in vitro* (Sassetti et al., 2003; Griffin et al., 2011; Reddy et al., 2014), and despite the fact that transcriptional silencing of *panK* (*coaA*) resulted in depletion of PanK protein to below the limit of detection, growth of *Mtb* in liquid culture and *in vivo* was not impeded (Reddy et al., 2014). A similar approach was used to show that >95% depletion of another enzyme in the CoA biosynthesis pathway, pantothenate synthetase (PanC), is required to effect complete growth inhibition of *Mtb in vitro* (Abrahams et al., 2012a). Likewise, in the case of the biotin biosynthesis enzyme, BioA, which is essential for growth *in vitro* and persistence of *Mtb* in mice (Sassetti et al., 2003; Woong Park et al., 2011), >95% depletion of the protein was required in order to completely inhibit *Mtb* growth (Woong Park et al., 2011). These results suggest that PanK, PanC, and BioA represent relatively invulnerable targets in *Mtb* and, since complete inactivation of a target’s function by a small molecule inhibitor is not readily achievable, this may account, at least in part, for the lack of whole-cell activity of known inhibitors of these enzymes against wildtype *Mtb* (Ciulli et al., 2008; Abrahams et al., 2012a; Bjorkelid et al., 2013; Reddy et al., 2014; Park et al., 2015). However, it is important to recognize that while knowledge of the relative vulnerabilities of potential targets – as inferred from target depletion studies – can be useful in terms of guiding target-based approaches to drug design, this notion is of less relevance in the case of an inhibitor that binds irreversibly to, or induces a conformational change in, its target. This point is illustrated in the case of BioA and the antibiotic, amiclenomycin: despite the relative invulnerability of BioA suggested by transcriptional silencing, amiclenomycin is able to bind covalently to this protein, thereby rendering it irreversibly inactive, which results in growth inhibition of *Mtb* (Sandmark et al., 2002; Woong Park et al., 2011).

## A Pathway Approach to Prioritizing Novel Drug Targets

There is renewed interest in targeting core metabolic pathways in *Mtb* for drug discovery (Murima et al., 2014; Puckett et al., 2014; Trujillo et al., 2014). In this context, the ability to rank enzymes in a prioritized metabolic or biosynthetic pathway on the basis of relative vulnerability can be particularly useful for focusing efforts. An excellent example of the value of this approach comes from a study on the biosynthesis of DPA, a precursor of the arabinogalactan component the mycobacterial cell wall (Kolly et al., 2014a). DPA biosynthesis in *Mtb* takes place in five steps involving eight genes (Wolucka, 2008; Kolly et al., 2014a), all but one of which are predicted to be required for bacillary growth (Sassetti et al., 2003; Griffin et al., 2011). The epimerase DprE1, which catalyzes the fifth and final step in the DPA biosynthesis pathway in conjunction with DprE2 (Wolucka, 2008; Kolly et al., 2014a), has been identified as the target of several new classes of inhibitors that display potent anti-tubercular activity, and has been rigorously validated as a novel TB drug target (Christophe et al., 2009; Makarov et al., 2009; Magnet et al., 2010; Stanley et al., 2012; Wang et al., 2013). To identify the steps in the DPA pathway that are most vulnerable to target depletion, Kolly et al. (2014a) generated conditional knockdown mutants in every gene and compared the effects of transcriptional silencing of each on the growth and viability of *Mtb in vitro* and in macrophages.

This study revealed that, in addition to *dprE1*, transcriptional silencing of *ubiA*, encoding the decaprenyl-phosphate phosphoribosyltransferase UbiA, and *prsA*, encoding the phosphoribosyl pyrophosphate (PRPP) synthetase PrsA, exhibited the most profound growth inhibitory effects, with depletion of each resulting in a loss of *Mtb* viability *in vitro* and in macrophages. While the cidality associated with transcriptional silencing of *drpE1* and *ubiA in vitro* was shown to occur as a result of induction of cell lysis, silencing of *prsA* resulted in reduced viability through cell implosion, without lysis occurring. Interestingly, although cell death occurred more slowly upon depletion of UbiA or PrsA than DprE1, the decrease in nucleic acid and protein synthesis observed as a consequence of PrsA depletion suggested that the bactericidal effects of PrsA inhibition are likely to be pleiotropic, thus identifying this enzyme as another promising a target in the DPA pathway. However, in an exciting new development, DprE1 was shown to occupy an extracytoplasmic location in *Mtb*, suggesting that cellular localization may be a major determinant of the vulnerability of DprE1 to chemical inhibition, accounting for the potency of the many inhibitors that act on this target (Brecik et al., 2015).

## Target Depletion by Tet-Mediated Regulated Protein Degradation

**FIGURE 4:**
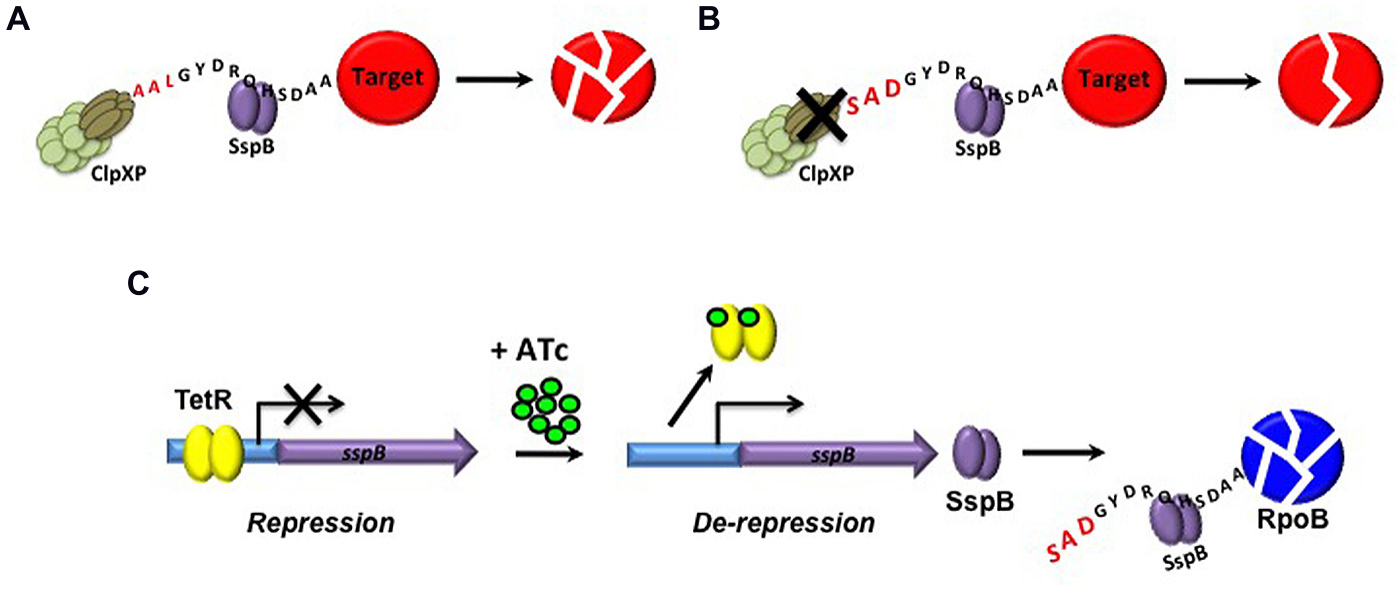
**Tetracycline-mediated regulated protein degradation in *Msm*. (A)** Degradation of SsrA-tagged proteins by ClpXP is enhanced by the adapter protein, SspB. **(B)** Mutation of the wildtype LAA ClpX recognition site of SsrA to generate a modified DAS tag weakens the interaction of ClpXP with the aberrant protein, resulting in SspB-dependent proteolysis of the tagged protein. **(C)** By replacement of the native *sspB* promoter with a Tet-inducible promoter, SspB-mediated degradation of proteins that have been engineered to carry a modified DAS tag can be achieved upon induction with ATc.

To overcome the limitations associated with using promoter replacement mutants for target depletion by transcriptional silencing, powerful new methods have been devised to enable regulated protein degradation in mycobacteria as an alternative approach (Kim et al., 2011; Wei et al., 2011). Although only reported thus far in *Msm*, this method nonetheless warrants discussion here owing to its obvious applicability in the validation of novel targets in *Mtb*. Proteolytic degradation complexes play an important role in protein quality control in bacteria by recognizing and degrading misfolded proteins. In *Mtb*, the ATP-dependent intracellular ClpXP proteolytic complex, which is essential for growth *in vitro* (Sassetti et al., 2003; Raju et al., 2012) and during infection (Raju et al., 2012), mediates degradation of SsrA-tagged proteins (**Figure 4A**; Raju et al., 2012). The recognition of aberrant proteins by ClpXP is further enhanced by binding of an adapter protein, SspB, to SsrA-tagged proteins (Levchenko et al., 2000). Mutation of the ClpX-binding region of the SsrA tag to generate a modified tag, DAS+4, which carries a terminal DAS sequence in place of the LAA sequence in wildtype SsrA, weakens the interaction between ClpX and the targeted protein, resulting in SspB-dependent proteolysis of the tagged protein (**Figure 4B**; Griffith and Grossman, 2008). Through elegant manipulation of the mycobacterial Clp protease system, Kim et al. (2011) demonstrated that degradation of DAS+4-tagged proteins could be induced by TetR-mediated induction of SspB in *Msm* (**Figure 4C**). Using this system, dose-dependent Tet-regulated depletion of RpoB was achieved by insertion of a DAS+4 tag at the 3′ end of the *rpoB* gene, which encodes the target of the first-line anti-TB drug, rifampicin. Importantly, targeted degradation of RpoB had the same bactericidal effect on *Msm* as treatment with rifampicin.

A variation of this approach was subsequently employed to compare the effects of regulated degradation of RpoB and five other well-validated antimycobacterial drug targets – GyrA, the target of the fluoroquinolones; Alr, a target of D-cycloserine; dihydrofolate reductase (DHFR), the target of trimethoprim; InhA, the target of isoniazid; and KasA, the target of thiolactomycin – on the growth and viability of *Msm* (Wei et al., 2011). This study revealed that the rates of depletion of the six targets varied considerably (Wei et al., 2011): while InhA, Alr, and DHFR were undetectable following 3 h of incubation in the presence of the inducer, little effect was seen on levels of RpoB, GyrA, and KasA up to 6 h post-treatment. Moreover, the degree of protein depletion observed did not correlate with bacterial survival: whereas 80% reduction of RpoB was sufficient to arrest the growth of *Msm*, >97% depletion of DHFR and Alr – as measured by immunoblotting and confirmed by enzyme activity analysis – had only modest effects on growth (Wei et al., 2011). Subsequent to this landmark study, regulated protein degradation has been use to validate a number of other drug targets in *Msm*, including those involved in nicotinamide adenine dinucleotide (NAD) biosynthesis (Rodionova et al., 2014), peptidoglycan biosynthesis (Gee et al., 2012) and intracellular protein degradation (Raju et al., 2012). Importantly, however, the varying effects of depleting pharmacologically validated targets of known TB drugs on *Mtb* viability calls for caution when using the outcome of regulated protein degradation studies as a primary or sole criterion for ranking, prioritizing or discarding novel drug targets.

## Tightened Regulation using a Tet-Mediated Dual Control System and its Application in the Validation of Persistence Targets

Most efforts in TB drug discovery remain centered on identifying inhibitors of cellular processes required for bacillary growth under various conditions (Makarov et al., 2009; Hartkoorn et al., 2012; Blanco et al., 2015; Manjunatha et al., 2015). However, the critical role that populations of bacilli that are non-replicating or slowly replicating are thought to play in TB disease (Barry et al., 2009; Rittershaus et al., 2013) has underscored the need to identify those cellular functions that are critical for maintaining these “persister” populations as well as inhibitors that act on them. Hypomorphs have a particularly important role to play in assessing the contribution of specific targets during different stages of disease progression, including the chronic stage, in which the rate of bacillary replication is markedly reduced (Gill et al., 2009). However, the relatively frequent generation of hypomorphs of *Mtb* in which growth is able to proceed unimpeded, even under conditions of maximal transcriptional silencing (Carroll et al., 2005, 2011; Woong Park et al., 2011; Kumar et al., 2012), can complicate the genetic validation of putative persistence targets *in vivo*. This problem is illustrated in the case a *bioA* hypomorph, which, despite the requirement of BioA for growth of *Mtb in vitro* (Sassetti et al., 2003; Woong Park et al., 2011) and in mouse spleen (Sassetti and Rubin, 2003), showed no growth phenotype when transcriptionally silenced *in vivo* owing to residual *bioA* expression at a level sufficient to sustain bacillary growth. In this example, mutation of the ribosome binding site upstream of *bioA* to create a weaker translation initiation signal resulted in dose-dependent growth of the *bioA* hypomorph *in vitro* and attenuation of growth in mouse lung (Woong Park et al., 2011), thus providing a strategy for overcoming the challenges associated with transcriptional leakiness.

**FIGURE 5:**
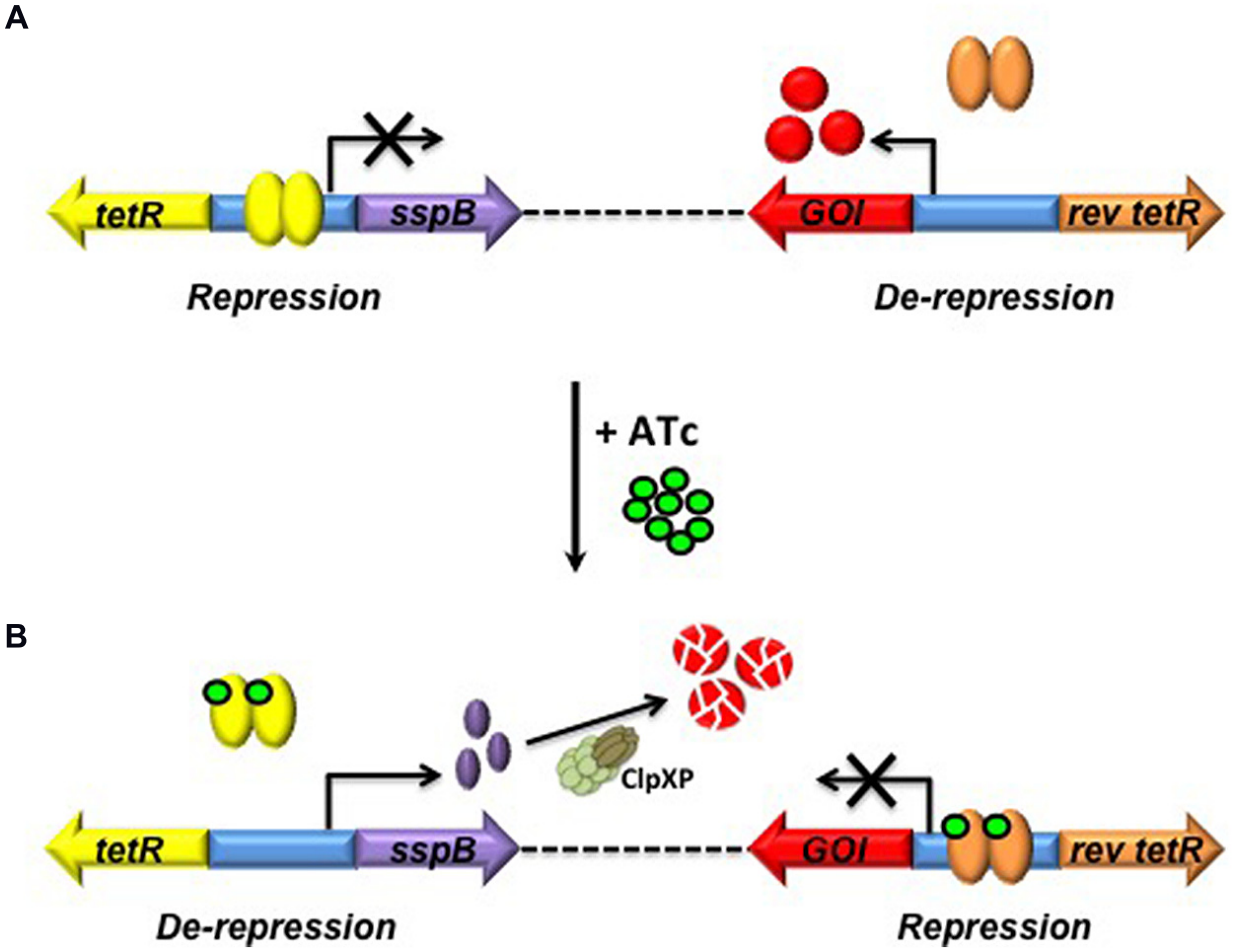
**Dual-control (DUC) genetic switch for simultaneous transcriptional silencing and degradation of the encoded protein in *Mtb*. (A)** In the absence of ATc *sspB* expression is repressed by TetR, while expression of the target gene under control of revTetR proceeds. **(B)** Upon introduction of ATc, *sspB* transcription proceeds, enabling ClpXP-mediated proteolysis of the target protein and simultaneous transcriptional repression of the target gene due to binding of revTet to the Tet-regulated promoter. *tetR*, Tet repressor; GOI, gene of interest; *rev tetR*, reverse Tet repressor; ATc, anhydrotetracycline; ClpXP, protease complex mediating controlled proteolysis of the target protein (Adapted from Kim et al., 2013).

An alternative strategy for addressing transcriptional leakiness is to use a modified version of the Tet-regulated system that allows simultaneous transcriptional repression of a target gene and degradation of the encoded protein, resulting in a more tightly regulated system (Kim et al., 2013; Kolly et al., 2014b). In the dual-control (DUC) system, expression of *sspB* is regulated by TetR, and is thus repressed in the absence of ATc, while expression of the target gene is regulated by revTetR, allowing it to be expressed in the absence of ATc (**Figure 5A**). Upon addition of the ATc inducer, transcription of *sspB* proceeds, leading to Clp-mediated proteolysis of the DAS+4-tagged target protein and simultaneous transcriptional silencing of the target gene through binding of revTetR to the Tet-regulated promoter (**Figure 5B**). The biological utility of the DUC system was confirmed by generating a BioA-DUC mutant, which showed faster kinetics of killing than achievable by transcriptional silencing or targeted proteolysis alone and also resulted in the reduced emergence of suppressors that were no longer inducer-responsive (Kim et al., 2013). A similar approach, using a modified version of the Pip/Tet-OFF regulated gene expression system (**Table 1**) in combination with SspB-mediated Clp proteolysis, was used to confirm the essentiality of the transketolase, Tkt, for growth of *Mtb* both *in vitro* and in macrophages (Kolly et al., 2014b). This system also demonstrated more rapid killing than transcriptional silencing alone (Kolly et al., 2014b), confirming the need to consider protein half-life when utilizing transcriptional silencing as a measure of target vulnerability.

Importantly, as control of the DUC system is Tet-mediated, it can also be applied *in vivo* to provide a powerful means of evaluating the requirement of a protein for persistence of *Mtb* during chronic infection, with reduced risk of suppressors masking the phenotype. Kim et al. (2013) used this approach to demonstrate the absolute requirement of NAD synthetase (NadE) for non-replicating persistence of *Mtb in vitro*, and to confirm that its inactivation is bactericidal. Furthermore, the rapid decline in bacillary load in the lungs and spleens of both acute and chronically infected mice upon inactivation of NadE indicated that inhibition of NadE could kill *Mtb* in several, if not all, of the metabolic states adopted during murine infection (Kim et al., 2013), further validating this enzyme as a drug target (Boshoff et al., 2008). The DUC genetic switch has also been used to validate fructose-1,6-bisphosphate aldolase (FBA), a central enzyme in glycolysis and gluconeogenesis in *Mtb*, as a persistence target (Puckett et al., 2014).

## Target-Based Whole-Cell Screening

An important application of hypomorphs is in target-based whole-cell screening (TB-WCS). The rationale underlying this approach is that a reduction in the concentration of a target may render the cell hypersensitive to inhibitors that act on that target. Firstly, hypomorphs can be applied in drug susceptibility testing of individual compounds to provide biological confirmation of target specificity. The utility of TB-WCS for confirming target specificity in *Mtb* was first demonstrated by the observation that dose-dependent silencing of *dprE1* resulted in hypersensitivity to benzothiazinones (Carroll et al., 2011), which are known inhibitors of DprE1 (Makarov et al., 2009). Subsequently, transcriptional silencing in *Mtb* has been used to confirm the target-specificity of PanC inhibitors designed through fragment-based approaches (Hung et al., 2009; Abrahams et al., 2012a); PanC (Kumar et al., 2013) and DHFR (Kumar et al., 2012) inhibitors identified from high-throughput biochemical screens; a LepB inhibitor previously shown to have increased efficacy against an *Escherichia coli lepB* knockdown (Barbosa et al., 2002; Ollinger et al., 2012a); and hydroxyurea, as an inhibitor of the class Ib ribonucleotide reductase (Singh et al., 2015). As outlined above, the discovery that the natural product, amiclenomycin, inhibits *Mtb* through covalent inactivation of BioA (Sandmark et al., 2002) has heightened interest in this particular target. However, amiclenomycin has significant liabilities, being chemically unstable, highly polar (Shi et al., 2011), and inactive against *Mtb* in in a mouse model of infection (Kitahara et al., 1975). To identify other scaffolds targeting BioA whose potency could potentially be enhanced by structure-based drug design to generate analogs with *in vivo* efficacy, Aldrich and colleagues (Park et al., 2015) used a BioA hypomorph to analyze the 255 hits identified by screening a 350,000-compound library for inhibitors of this enzyme. Their results were encouraging: while the majority of hits identified from the biochemical screen showed no whole-cell activity against *Mtb*, several showed enhanced activity against the BioA hypomorph, confirming that these compounds were on-target in *Mtb* (Park et al., 2015).

Target-based whole-cell screening has also found application in enabling the direct identification of novel, cell-permeable inhibitory scaffolds with whole-cell activity against defined targets or pathways through screening of large compound libraries. The hits may include inhibitors of non-catalytic sites in an enzyme target. They may also include chemotypes that would escape detection in screens against wildtype cells by virtue of their weak inhibitory activity, thus increasing the availability of chemical matter for lead identification (**Figure 3B**). The feasibility of TB-WCS as a tool for identifying novel antibacterial agents was first demonstrated by the discovery of the type II fatty acid synthesis (FASII) inhibitor, platensimycin, which showed enhanced activity against *Staphylococcus aureus* isolates in which *fabF* transcript was conditionally depleted by antisense-mediated interference (Wang et al., 2006; Young et al., 2006). Although TB-WCS has been used with some success in other bacteria (DeVito et al., 2002; Wang et al., 2006; Young et al., 2006; Phillips et al., 2011), it has only recently been applied in *Mtb* (Abrahams et al., 2012a). In the only example published to date, a *panC* hypomorph was screened against a ∼14,000-compound library which led to the identification of a series of flavones that displayed increased potency against PanC-depleted cells relative to wildtype *Mtb*. It was subsequently shown that these compounds do not directly inhibit PanC, despite the fact that pantothenate – the product of the PanC enzyme – rescued *Mtb* from the toxic effects of the compounds (Abrahams et al., 2012a). This result confirmed that the effect of target depletion can extend beyond hypersensitization of the target itself, and may, for example, hypersensitize downstream enzymes in the same metabolic pathway to chemical inhibition through substrate depletion or diversion of metabolic flux (Palmer and Kishony, 2014). In the case of PanC, the potential for off-target hypersensitization owing to metabolic remodeling is even greater, as depletion of this target leads to CoA depletion, which may, in turn, result in hypersensitization of any number of CoA-dependent enzymes. Target de-convolution in cases such as this may be challenging.

In addition to this potential complication, there are a number of other issues that should be taken into account when considering the use of TB-WCS for hit identification in TB drug discovery. Firstly, it is critical to monitor and confirm stability of the hypomorph during the course of the phenotypic screen as the emergence of suppressors that are no longer inducer-dependent will compromise the screen. Secondly, as highlighted recently (Palmer and Kishony, 2014), an alteration in the level of expression of a target does not always manifest in a predictably altered susceptibility to a drug that acts on that target, particularly for cases in which the mechanism of action is complex. The fluoroquinolones provide an interesting case in point: these drugs act by trapping gyrase-DNA complexes, resulting in inhibition of DNA synthesis and growth arrest, followed by cell death upon release of double-stranded DNA breaks from trapped gyrase complexes (Drlica and Zhao, 1997). As such, conditional depletion of GyrA is not expected to confer fluoroquinolone hypersensitivity on mycobacteria (Wei et al., 2011). Finally, as for any phenotypic screen, the differential requirement of genes for growth under specific conditions (Pethe et al., 2010; Mak et al., 2012; Beste et al., 2013) must also be taken into account for TB-WCS. As an example, transcriptional silencing of *icl1* resulted in Tet-dependent growth impairment of *Mtb* on acetate as the sole carbon source and hypersensitivity to 3-NP, but had no effect on growth of 3-NP susceptibility when cultured on glucose.

## Limitations in using Hypomorphs to Simulate Chemical Inhibition

While the utility of hypomorphs in TB drug discovery has been convincingly demonstrated, variability between individual targets can sometimes make data interpretation difficult. Furthermore, the concept of genetic validation is predicated on the assumption that a perturbation in the cellular concentration of a target by transcriptional silencing or proteolytic degradation is a reasonable surrogate for the inhibitory effect of a chemical inhibitor of the target’s function. In reality, the genetic vs. chemical vulnerability of a target may differ considerably as a result of a variety of factors (Wei et al., 2011). These include the local concentration of drug required for effective inhibition of the target and the time needed for the successful perturbation of the target (Lu and Tonge, 2010), both of which are likely to relate to the abundance of the target. The former is of particular significance in the case of *Mtb*, whose sequestration in remote lesion compartments in the human host likely accounts for the lack of correlation observed between plasma pharmacokinetics and drug efficacy (Kjellsson et al., 2012; Lakshminarayana et al., 2015). The latter is important with regard to using genetic approaches to simulate chemical inhibition since the kinetics of chemical inhibition are significantly faster than those of gene silencing. While targeted protein degradation (Kim et al., 2011, 2013; Wei et al., 2011; Kolly et al., 2014b) has the potential to more accurately estimate target vulnerability by better simulating the kinetics of chemical inhibition (Kim et al., 2011, 2013; Kolly et al., 2014b), this approach has its own limitations: in particular, it involves modification of the C-terminus of the target protein, which could affect the structure, function and abundance of the target. The recent development of a clustered regularly interspaced short palindromic repeat interference (CRISPRi) approach to regulating gene expression in *Mtb* has the potential to overcome these limitations in that the transcriptional level of the target is maintained no modification of the expressed protein is required (Choudhary et al., 2015).

The potential disconnect between depletion of a target by genetic means and its pharmacologic inhibition is perhaps best illustrated in the case of DHFR, the target of the folate biosynthesis inhibitor, trimethoprim. Although overexpression of DHFR in both *E. coli* (Palmer and Kishony, 2014) and *Msm* (Bhat et al., 2013) was shown to result in decreased susceptibility to trimethoprim, targeted depletion of DHFR by proteolytic degradation in *Msm* did not phenocopy treatment with trimethoprim (Wei et al., 2011). One possible explanation is that chemical inhibition of DHFR by trimethoprim is more efficient than targeted proteolysis. Alternatively, trimethoprim could have multiple targets, requiring simultaneous inhibition of all in order to mediate growth inhibition. To distinguish these possibilities, the metabolomic consequences of DHFR depletion and treatment with sub-inhibitory concentrations of trimethoprim were compared. The production of the same metabolite profiles in both cases confirmed that DHFR is in fact the primary target of trimethoprim (Wei et al., 2011) suggesting that, in this case, even regulated degradation of the DHFR is not able to accurately recapitulate the phenotypic consequences of chemical inhibition of the enzyme.

## Concluding Remarks

Given that less than a decade ago there were no robust genetic switches available for regulation of gene expression in *Mtb*, remarkable progress has been made in this area. Hypomorphs provide an effective means of genetically validating novel drug targets, of comparing selected mutants in terms of vulnerability and of evaluating the downstream consequences of loss of target function. However, other factors such as cellular localization and druggability, which are critically important in determining target access by small molecule inhibitors, must also be taken into account when ranking and prioritizing novel drug targets, particularly in a pathway context. The implementation of regulated protein degradation systems and DUC genetic switches provide enhanced stringency while simultaneously enabling more rapid kinetics of inactivation, thus providing a powerful means of validating new drug targets. The increasing accessibility of ‘omics approaches offer exciting prospects for combining metabolomic and proteomic analyses with genetic approaches for target validation, which, when coupled with live-cell imaging of bacilli undergoing target depletion or drug treatment, have the potential to provide key insights into the global effects that disruption of an essential cellular function can have upon bacterial metabolism and physiology. Whether the systems reviewed here have a role in validating targets in animal models other than mice, which more accurately recapitulate the pathology of human disease, remains to be seen. Nonetheless, the encouraging results obtained to date, suggest that conditional mutants are set to play an increasingly important role in early stage TB drug discovery.

## Conflict of Interest Statement

The authors declare that the research was conducted in the absence of any commercial or financial relationships that could be construed as a potential conflict of interest.
